# The return to work after arthroplasty (REWARD) cohort: a nationwide longitudinal study of sick leave and disability pension among individuals receiving hip and knee arthroplasty in Sweden

**DOI:** 10.1136/bmjopen-2026-119123

**Published:** 2026-07-15

**Authors:** Kristin Gustafsson, Per Liv, Ola Rolfson, P Paul F M Kuijer, Sebastian Mukka, Jens Wahlström

**Affiliations:** 1 Department of Health, Medicine and Caring Sciences, Linköping University, Linköping, Sweden; 2 Rehabilitation Centre, Ryhov County Hospital, Jönköping, Sweden; 3 Department of Public Health and Clinical Medicine, Umeå University, Umeå, Sweden; 4 Institute of Clinical Sciences, Sahlgrenska Academy, University of Gothenburg, Gothenburg, Sweden; 5 Orthopaedics, Sahlgrenska University Hospital, Gothenburg, Sweden; 6 Department of Public and Occupational Health, Amsterdam UMC, University of Amsterdam, Amsterdam, the Netherlands; 7 Department of Diagnostics and Intervention, Umeå University, Umeå, Sweden; 8 Department of Epidemiology and Global Health, Umeå university, Umeå, Sweden

**Keywords:** Occupational Health Services, Orthopedics, EPIDEMIOLOGIC STUDIES, REGISTRIES

## Abstract

**Abstract:**

**Purpose:**

The return to work after arthroplasty (REWARD) study was established to address knowledge gaps regarding factors influencing return to work and long-term workability following hip and knee arthroplasty. The study investigates how variables such as sex, occupational workload and prior treatments affect sick leave patterns across regions and healthcare providers in Sweden. By analysing trajectories of sick leave and disability pension before and after surgery, the study aims to identify determinants for successful return to work.

**Participants:**

Initially, we identified two separate cohorts comprising all individuals registered in the Swedish Arthroplasty Register who received a primary hip or knee arthroplasty between 2012 and 2022. Eligible individuals were 18–69 years of age and had osteoarthritis (OA) as the indication for surgery. For each of the arthroplasty cohorts, Statistics Sweden created a reference cohort by randomly selecting three individuals from the general population for each arthroplasty case, matched on age, sex and municipality.

**Findings to date:**

Using linked nationwide registers, we identified 64 370 individuals who received primary hip arthroplasty and 60 683 who received knee arthroplasty during 2012–2022, all aged 18–69 years and diagnosed with OA. Median age was 62 years (IQR 56–66) for hip and 63 years (IQR 58–66) for knee arthroplasty; 52% and 55% were women respectively.

The corresponding matched cohorts consisted of 193 110 and 182 049 individuals respectively.

The dataset includes patient and surgical characteristics, 1-year patient-reported outcomes, first-line treatment participation, socioeconomic data, occupational exposures and sick leave and disability pension data (5 years before and after surgery). Data collection is complete.

**Future plans:**

The REWARD cohort is intended for longitudinal analysis of return to work and long-term workability following hip and knee arthroplasty, and for quantifying associated societal costs. Data collection is anticipated to be completed in 2027, pending potential linkage of comorbidity and prescribed medication data. Findings from REWARD will inform national guidelines and decision support tools for sick leave and work-related rehabilitation. Future work includes co-developing individualised, work-oriented interventions in collaboration with patients and healthcare professionals.

**Strengths and limitations of this study**

- The Return to Work After Arthroplasty study will use nationwide population-based cohorts to examine patterns of sick leave and work disability among patients receiving hip and knee arthroplasty, respectively.
- A reference cohort from the general Swedish population, matched by age, sex and municipality of residence, will be included for comparative purposes.
- By identifying key determinants of work participation through trajectories of sick leave and disability pension, the study highlights opportunities for targeted interventions to enhance recovery and long-term work participation.
- Differences in sick leave policy between countries may limit the generalisability of findings.
- Limited availability of data on comorbidities in both cohorts and lifestyle factors (eg, smoking and physical activity) in the reference cohort restricts interpretation and comparison.

## Introduction

 Osteoarthritis (OA) is the most common form of arthritis and a leading cause of disability worldwide.^1^ The hip or knee is commonly affected, leading to gradually increasing joint pain and functional limitations.^2^ First-line treatment typically includes patient education, exercise therapy and weight control if needed.^3^ For individuals with advanced disease progression, joint arthroplasty may be indicated to reduce pain and improve function and quality of life.^3^

Due to demographic changes and an ageing population, the incidence of hip and knee arthroplasties has increased over the past decade and is expected to continue rising.^4 5^ Moreover, the number of procedures among younger individuals is also growing,^6 7^ partly due to advances in surgical techniques and implant design that have improved longevity.^8 9^ This trend places increased demands on supporting sustainable working lives.^10 11^

A successful return to work (RTW) and long-term workability after surgery are essential to prevent prolonged sick leave periods and early retirement, regardless of the type of work performed. However, there is limited knowledge about the factors influencing RTW and sustained work participation following surgery.^12^ For example, previous studies have demonstrated prolonged sickness absence and sex differences in RTW but have lacked detailed data on occupational exposures and disability pension.^13^ Moreover, no nationwide study has examined longitudinal sick-leave trajectories and transitions to disability pension following arthroplasty. This knowledge gap may contribute to regional and provider-level variations in sick leave patterns.

The return to work after arthroplasty (REWARD) study will describe and analyse sick leave patterns before and after hip and knee arthroplasty and compare them to those of the general population. By identifying factors associated with RTW—such as sex, occupational workload, healthcare provider and previous treatments—we aim to develop strategies to optimise and individualise vocational rehabilitation.

## Cohort description

The REWARD study comprises nationwide cohorts of individuals receiving hip and knee arthroplasty for OA, along with matched reference populations.

### Study design

This nationwide observational cohort study is based on register data from the Swedish national quality registers: the Swedish Arthroplasty Register^14^ and the Swedish Osteoarthritis Register.^15^ Additional individual-level data were obtained from Statistics Sweden and the Swedish Social Insurance Agency (SSIA). Data linkage was performed using the unique personal identity number (PIN) assigned to all Swedish residents at birth or on registration as a permanent resident.^16^ The datasets were delivered to the research team in January 2025.

### Study population

**Figure 1:**
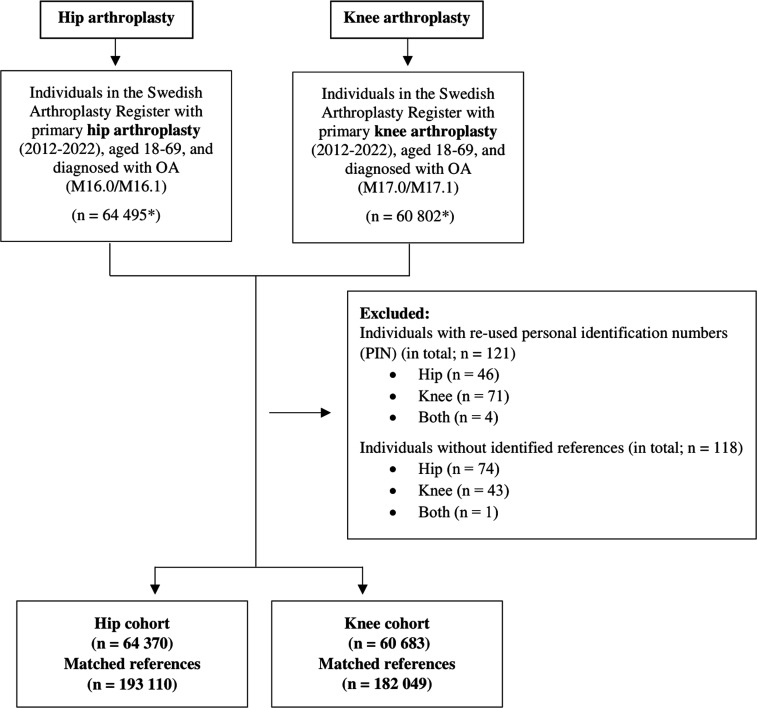
Flow chart of the inclusion and selection of the study population. ^*^Individuals who received both procedures were included in both cohorts. OA, osteoarthritis.

The study population included all individuals aged between 18 and 69 years who received a primary hip or knee arthroplasty due to OA (ICD10 codes M16.0, M16.1, M17.0 and M17.1) between 2012 and 2022, as recorded in the Swedish Arthroplasty Register. Patients were categorised into a hip and a knee arthroplasty cohort (figure 1), where individuals who received both procedures were included in both cohorts. For each patient, Statistics Sweden randomly selected three reference individuals from the general population, matched by age, sex and municipality at time of surgery.

### Data sources and data collection

#### The Swedish Arthroplasty Register

The Swedish Arthroplasty Register captures data on patient demographics, surgical procedures, surgical centre characteristics (both public and private) and patient-reported outcomes preoperatively and 1 year postoperatively (see table 1 for description of collected variables). The completeness of primary surgeries reported to the Swedish Arthroplasty Register is 98.5% for hips and 97.5% for knee arthroplasties.^17^

**Table 1:** Overview of the variables from the Swedish Arthroplasty Register and the Swedish Osteoarthritis Register used in the REWARD study

The Swedish Arthroplasty Register		The Swedish Osteoarthritis Register	
Variable	Comment	Variable	Comment
**Patient characteristics preoperatively**		**Patient-reported data reported before intervention and at 3-month follow-up**	
Age		Weight/height/BMI*	
Sex		Other affected joints*	
ASA Physical Status	Preoperative physical status classification (I–V), ranging from I: healthy patient to V: not expected to survive without surgery	Walking difficulties	
Weight/height/BMI		Charnley class	Classifications of musculoskeletal impairment (A–C); A: unilateral hip or knee affected, B: bilateral hip or knee affected and C: multiple joints affected or other condition affecting mobility.
Diagnosis	ICD10 codes: M16.0, M16.1, M17.0, M17.1	Desire for surgery	
Previous surgeries†		Fear of movement	
**Primary surgical procedure**		Pain in most affected joint	Including pain frequency and pain intensity
Surgical date		EQ5D-5L	Measure of health-related quality of life
Prosthesis type and fixation	To define total or partial prosthesis and type of fixation	Physical activities/training	Weekly
Side		Sick leave*	Due to joint problems
Surgical approach‡		Pain persistent after intervention§	
**Patient-reported factors preop and 1 year postop**		**Physiotherapist-reported data before intervention and at 3-month follow-up**	
Smoking¶		Visit date	
Joint pain	Reported for the last 4 weeks, in the right and left side, respectively	Most affected joint	Hip or knee
Physical activity/training	Weekly	Worst side	
Charnley class	Classifications of musculoskeletal impairment (A–C); A: unilateral hip or knee affected, B: bilateral hip or knee affected and C: multiple joints affected or other condition affecting mobility	Previous examinations*	Including radiography and MRI in the most affected or the contralateral joint
EQ5D-5L	Measure of health-related quality of life	Previous physiotherapy/first-line intervention*	Including physiotherapy, information on adapted training and information on weight reduction
Previous physiotherapy/ first-line intervention‡¶		Previous other treatments*	Including OA-related drugs and surgery in the most affected or contralateral joint
Satisfaction**		Description of participation in first-line intervention§	Including assessment, patient education and exercise therapy
**Surgical centre characteristics**		Waiting list for arthroplasty*	
County	Based on the existing 21 counties in Sweden		
Hospital	Performing arthroplasty surgery in Sweden		
Type of hospital	University, other public or private		
**Re-surgery procedure**			
Re-surgery date			
Re-surgery type	Replacement, extraction or other type of procedures		
Re-surgery diagnosis			

Detailed information regarding each variable is available in Swedish on the receptive registry’s website (https://slr.registercentrum.se/ and https://boa.registercentrum.se/).

* Only before start of the first-line intervention.

† Only registered for knee arthroplasty.

‡ Only registered for hip arthroplasty.

§ Only at the evaluation 3 months after the first-line intervention

¶ Only reported preoperative.

** Only reported postoperative.

ASA Physical Status, American Society of Anesthesiologists Physical Status; BMI, body mass index; EQ5D-5L, EuroQol five-dimenstion five-level index questionnaire; ICD10, International Classification of Diseases, 10th revision; OA, osteoarthritis.

#### The Swedish Osteoarthritis Register

The Swedish Osteoarthritis Register captures data on first-line interventions for hip and knee OA (eg, patient education and exercise therapy) from approximately 780 physiotherapy units in primary healthcare.^18^ Data include symptom severity, intervention type and adherence (see table 1 for description of collected variables).

#### Statistics Sweden

Statistics Sweden is the national governmental agency responsible for producing official statistics. Data are collected from administrative registers and national surveys. For this study, we used two key sources:

- *The Swedish Total Population Register* was used to identify reference individuals and to obtain data on birth year, sex, country of birth, municipality of residence, emigration and death.^19^
- *The Longitudinal Integration Database for Health Insurance and Labour Market Studies (LISA)* provided annual data on civil status, educational attainment, employment status, occupation and disposable income (see table 2 for description of data delivery and handling).^20^

**Table 2:** Description on data delivery from Statistics Sweden and data handling in the creation of the REWARD research database

Statistics Sweden		
Indicator	Description of delivered data	Data handling in creation of the database
**The Swedish Population Register**		
Year of birth		
Sex	Man/woman	
Municipality	Based on the 290 municipalities in Sweden	
Region	Based on the 21 regions in Sweden	
Emigration		
Country of birth	Delivered in 11 groups of countries: Sweden; Nordic countries (other than Sweden); EU28 (except the Nordic countries); Europe (except EU28 and the Nordic countries); Africa; North America; South America; Asia; Oceania; Soviet Union (when existing); unknown	
Date of death		
**The Longitudinal Integration Database for Health Insurance and Labour Market Studies (LISA)**		
Civil status	Delivered, coded as: married (G); unmarried (OG); registered partner (RP); divorced (S); divorced partner (SP); widow/widower (Ä); surviving partner (EP)	
Educational attainment	Educational level is classified using SUN (Swedish Educational Terminology), which is based on the international classification ISCED (International Standard Classification of Education). SUN consists of two modules: a level module and an alignment module In 2019, SUN2000 was replaced by SUN2020, with no changes made to the level module	We used the level module, which applies a 3-digit code to classify the level, length and type of education. The highest educational level was categorised into seven aggregated groups: 1=not completed compulsory (<9 years); 2=completed compulsory (9 years); 3=upper secondary (≤2 years); 4=upper secondary (3 years); 5=post-secondary (<3 years, eg, vocational diplomas); 6=post-secondary (≥3 years, eg, bachelor’s/master’s/professional degrees); 7=research education (licentiate/PhD)
Employment	In LISA, employment status is derived from multiple sources, primarily employer-reported earnings and self-employment tax records. A model-based income threshold defines employment, and certain social insurance benefits (eg, sickness, parental) also qualify as employment	During the study period (2012–2002, including 5 years before and after surgery), Statistics Sweden revised its employment classification, adjusting the upper age limit and the classification of self-employed individuals aged 65 and older (retirement age). These changes had minor effects on our cohort To ensure consistency, we harmonised the variables into a single employment indicator based on the following sources: SyssStatJ (2007–2010), SyssStat11 (2011–2018), SyssStat19 (2019–2021) and BASAlt (2022). Employment status was coded according to the criteria in the respective variables as follows: 1=Employed (aged 16–84 in SyssStatJ; aged 16–74 in SyssStat11 and SyssStat19; also aged ≥75 in SyssStat19; and as register-based employed in BASAlt) 2=Unemployed (with or without reported income in SyssStat; and as register-based unemployed in BASAlt)
Disposable income	Defined as total income after taxes and negative transfers. It includes earnings from employment, pensions, sickness and parental benefits, other social transfers (via SSIA), as well as income from business and capital, net of taxes and deductions. To enable comparisons across households, Statistics Sweden calculates disposable income per consumption unit using an equivalence scale (DispInkKE04), assigning weights: 1.00 for the first adult, 0.51 for the second adult, 0.52 for the first child (0–19 years) and 0.42 for each additional child. For example, a household with two adults and two children has a weight of 2.45^20^	In the REWARD project, disposable income per consumption unit will be reported and converted to the appropriate currency depending on the target journal
Occupation	Occupation data in LISA is sourced from the Swedish Occupational Register, using three-digit (Ssyk3) or four-digit (Ssyk4) codes Occupations are classified according to Sssyk96 until 2013, and Ssyk2012 from 2014 onwards In both systems, the first digit represents one of ten major occupational groups, based on type of work, tasks and required level of skills	Depending on the research question and required level of detail, we will assess whether the classification systems can be merged using existing translation keys or handled separately. Occupation will also be used in combination with SweJEM to estimate physical and psychosocial occupational exposures

REWARD, Return to Work After Arthroplasty; SSIA, the Swedish Social Insurance Agency; SweJEM, Swedish Job Exposure Matrix.

Data from Statistics Sweden were collected for all individuals in the hip and knee arthroplasty cohorts and their matched references, covering a 10-year period: 5 years before and 5 years after surgery. These data enabled longitudinal characterisation of both patients and reference individuals in terms of demographic and socioeconomic factors, and were also used to link occupational codes to the Swedish Job Exposure Matrix (SweJEM).^21^

### The Swedish Social Insurance Agency

The SSIA administers Sweden’s public social insurance system, including sickness benefits and disability pensions. They host the MiDAS database, which contains detailed records of all social insurance claims.

In Sweden, individuals who are legally employed or registered as unemployed are entitled to sickness benefits from day 2 of a reported sickness period. For employed individuals, benefits from day 2 to day 14 are paid by the employer. If the sickness period exceeds 14 days, the SSIA takes over responsibility for payment starting from day 15. Sickness absence longer than 7 days must be certified by a physician. Absences of 1–14 days are not registered by SSIA, but longer periods are recorded in MiDAS.

From MiDAS, we retrieved information on sickness absence spells, including start and end dates, extent (full or part-time), diagnosis and disability pension status. Data are available for all individuals in the hip and knee arthroplasty cohorts and their matched references, covering a 10-year period: 5 years before and 5 years after surgery. These data enable analysis of patterns in work absence and long-term work incapacity.

#### Swedish Job Exposure Matrix

The SweJEM is a national job exposure matrix, developed at the Karolinska Institute (Sweden) and is based on self-reported exposure data from the Swedish Work Environment Survey (1997–2013).^21^ We will use SweJEM to estimate physical and psychosocial occupational exposures based on occupational codes from the LISA register.

### Exposure and potential confounders

Key exposures include occupational exposure (assessed via SweJEM), sex, age, healthcare provider and region. Potential confounders include socioeconomic status. Detailed variable definitions are presented in tables1  2.

### Patient and public involvement

Patients and members of the public were not involved in the planning, design or conduct of the initial phase of this study. However, patient and stakeholder involvement is planned for the second phase of the REWARD project. A reference group will be established, including representatives from patient organisations, healthcare professionals, occupational health services and the SSIA.

This reference group will participate in structured collaboration workshops aimed at supporting the dissemination and practical application of research findings within Swedish healthcare and working life. Future activities also include co-developing individualised, work-oriented interventions in partnership with patients and clinical stakeholders.

## Findings to date

In total, we identified 64 370 individuals who received a primary hip arthroplasty between 2012 and 2022, were 18–69 years of age and had an OA diagnosis (M16.0/M16.1), and 60 683 who had received a primary knee arthroplasty during the same period, in the same age range, with OA as the diagnosis (M17.0/M17.1). Both cohorts included data from all 21 Swedish regions. The corresponding reference cohorts included 193 110 and 182 049 individuals, respectively (figure 1).

In the hip arthroplasty cohort, the median age was 62 years (IQR 56–66), with 52% women and a median body mass index (BMI) of 27.5 kg/m^2^ (IQR 24.8–30.8). In the knee arthroplasty cohort, the median age was 63 years (IQR 58–66), 55% were women, and the median BMI was 29.1 kg/m^2^ (IQR 26.3–32.4).

Of the arthroplasty cases across the hip and knee cohorts, 3673 (3%) individuals were identified in both cohorts. In the hip cohort, 11 187 (17%) had received bilateral hip arthroplasties during the study period, of which 490 (4%) were performed on the same day. In the knee cohort, the corresponding numbers were 14 076 (23%) bilateral knee arthroplasties during the study period, of which 1605 (11%) were performed on the same day. Re-surgeries during the study period were performed among 2637 (4%) individuals of the hip cohort and 3530 (6%) individuals of the knee cohort.

Of all unique individuals in the hip and knee cohorts, 21 058 (17%) also had a registration in the Swedish Osteoarthritis Register, indicating participation in first-line OA treatment prior to their primary arthroplasty.

### Ongoing and planned studies

Several studies are currently being initiated based on the REWARD study population. The initial focus is on analysing patterns of sick leave before and after hip and knee arthroplasty, using data from SSIA (MiDAS) and Statistics Sweden. These patterns will be further examined in relation to factors such as sex, geographic region, surgical centres characteristics, occupational physical workload (via SweJEM) and prior participation in first-line interventions for OA (from the Swedish Osteoarthritis Register).

In addition, sick leave and disability pension outcomes will be compared with those of the matched reference cohort from the general Swedish population, to identify risk factors associated with sustainable RTW and receipt of disability benefits.

We also plan to develop predictive models to estimate the economic burden of sick leave and disability pensions associated with OA-related arthroplasty, projecting trends up to the year 2050. Additionally, we will investigate the feasibility of integrating data on comorbidity and prescribed medications into the REWARD research database.

In the final phase of the project, insights from these analyses will be used to inform the development of future interventions aimed at optimising RTW after hip and knee arthroplasty. This work will be conducted in collaboration with healthcare professionals, patient representatives and other relevant stakeholders, and will also draw on findings from additional qualitative research.

### Strengths and limitations

The REWARD study has several notable strengths. First, it includes a nationwide cohort of individuals aged 18–69 years who received primary hip or knee arthroplasty due to OA over an extended period of 11 years. Only those with primary hip and knee OA were included to ensure a more homogeneous and interpretable study population. The Swedish Arthroplasty Register, which captures 97%–98% of all primary arthroplasty procedures in Sweden,^17^ ensures near-complete inclusion of eligible patients.

Using Sweden’s unique PIN, we were able to link individual-level data across multiple national registers, allowing adjustment of several potential confounding factors. Loss to follow-up is minimal due to the administrative nature and continuous updates of these registers.^19^

A key strength of the REWARD cohort is its multi-dimensional design, combining surgical data, occupational exposure (via SweJEM), socioeconomic information (via Statistics Sweden) and social insurance outcomes (via SSIA). This integration enables comprehensive analyses of factors influencing recovery and RTW after arthroplasty. The inclusion of a matched reference cohort from the general population further strengthens the study’s ability to identify risk factors and contextualise outcomes.

The creation of the REWARD cohort highlighted the value of Sweden’s register infrastructure for longitudinal research. The ability to link high-quality data across health, social insurance and labour market domains was essential for the study’s design and scope. This linkage will enable comprehensive analyses of sick leave patterns and factors influencing RTW, which are central to the REWARD study’s objectives. In the long term, these analyses can inform strategies to optimise and individualise vocational rehabilitation, enhance clinical care to facilitate work participation after arthroplasty and contribute to the development of national guidelines and decision-support tools for sick leave and work-related rehabilitation.

However, the study also has limitations. We lack detailed medical data on comorbidities, which may act as important confounders. Although American Society of Anesthesiologists (ASA) Physical Status and Charnley score are available for the arthroplasty cohorts, these are coarse measures and are not available for the matched reference populations. Therefore, we will investigate the feasibility of integrating data on comorbidity as well as prescribed medications into the REWARD research database for both the arthroplasty cohorts and their references. Similarly, BMI data are only available for the arthroplasty cohorts, limiting direct comparison. We also lack data on lifestyle factors such as physical activity and smoking for all participants.

Furthermore, limitations related to register-based data should be considered. Misclassification of exposures and outcomes may occur, as register data are not primarily collected for research purposes. For example, ICD-10 coding in the SSIA register is incomplete, limiting the ability to distinguish sick leave specifically related to OA or surgery. While subgroup analyses may be possible for individuals with recorded OA diagnoses, most analyses will focus on all-cause sick leave. In addition, sick leave for periods shorter than 14 days is not registered and will therefore not be possible to study. Missing data may also affect the analyses and should be addressed in the analyses.

In addition, the use of job-exposure matrices (JEMs), such as SweJEM, introduces exposure misclassification, as estimates are based on occupational groups rather than individual-level measurements. JEMs are subject to Berkson-type measurement errors, which do typically not introduce systematic attenuation of estimated associations in linear analyses, unlike classical measurement error, but increased variance and wider CI estimates. However, in non-linear models, such as Cox regression, some attenuation of effect estimates (bias toward the null) remains possible.^22^ There is also a potential risk of bias related to cohort structure, as some individuals may contribute to both the hip and knee arthroplasty cohorts (eg, if receiving both procedures), which will need to be carefully accounted for in future analyses.

Finally, differences in sick leave policies and social insurance systems between countries may limit the generalisability of findings beyond the Swedish context. Variations in eligibility criteria, compensation levels and administrative procedures could influence both the duration and reporting of sick leave, which should be considered when interpreting results internationally.

## Data Availability

Data are available upon reasonable request.
