# Supplementary material for: The return to work after arthroplasty (REWARD) cohort: a nationwide longitudinal study of sick leave and disability pension among individuals receiving hip and knee arthroplasty in Sweden
Source: BMJ Open. 2026 Jul 15;16(7):e119123. doi: 10.1136/bmjopen-2026-119123 (PMC13374432; doi:10.1136/bmjopen-2026-119123)
Supplement: Supplementary file 1 [file bmjopen-2026-119123.reviewer_comments.pdf]

PEER REVIEW HISTORY

BMJ Open publishes all reviews undertaken for accepted manuscripts. Reviewers are asked to complete a checklist review form and are provided with free text boxes to elaborate on their assessment. These free text comments are reproduced below.

ARTICLE DETAILS

Title (Provisional)

Cohort profile: the Return to Work After Arthroplasty (REWARD) cohort, a nationwide longitudinal study of sick leave and disability pension among individuals receiving hip and knee arthroplasty in Sweden

Authors

Gustafsson, Kristin; Liv, Per; Rolfson, Ola; Kuijer, P. Paul F.M.; Mukka, Sebastian; Wahlström, Jens

---

VERSION 1 - REVIEW

**Reviewer**	**1**
**Name**	**Boonen, Bert**
**Affiliation**	**Zuyderland Medical Center**
**Date**	**08-Apr-2026**
**COI**	**None**

Comments to the Author

Thank you for the opportunity to review this well constructed cohort profile. The manuscript presents a valuable and ambitious nationwide resource for studying sick leave and work disability following hip and knee arthroplasty in Sweden. The rationale is clear, the cohort is comprehensively described, and the linkage of multiple high-quality national registers is a major strength. The paper is generally well written and easy to follow.

In addition to several minor suggestions for clarity, there are a few points where the reviewer form required a no response. These do not reflect major flaws but indicate areas where the manuscript could strengthened.

General comments

The manuscript is clear, well structured and presents a valuable nationwide cohort. A few areas could be refined for clarity and conciseness:

Abstract Well written; consider slightly shortening the *Findings to date* section to highlight only the key cohort characteristics.

Introduction Strong and well motivated; you may further emphasise the specific gaps in the existing literature on return to work.

Cohort descripion Comprehensive and transparent; a brief orienting summary at the start could improve readability.

Finding Informative but could be more concise to better match the purpose of a cohort profile.

Collaboraion section Helpful, though it could be more specific regarding types of collaborations and include a clearer data availability statement.

With minor revisions to clarity and completeness, the manuscript will be well suited for publication.

Points marked no in the reviewer form

7. Statistics appropriate and fully described? no

The manuscript staes that several analyses are planned but does not describe any statistical methods. For a cohort profile this is acceptable, but since the reviewer form asks specifically whether statistical methods are appropriate and fully described,t answer is No. The paper outlines what will be analysed but not how.

No statistical methods are described anywhere in the manuscript. Specifically, the manuscript does not indicate: how sick leave trajectories will be modelled, how determinants of return to work will be analysed, how confounding will be handled, how missing data will be addressed, whether time-to-event, mixed models, or trajectory modelling will be used, how comparisons with the reference cohort will be performed.

There is no Statistical analysis section at all. A brief outline of planned analytical approaches would address this.

9. Do the results address the research question? no

The manuscript presents only descriptive cohort characteristics. This is appropriate for a cohort profile, but it does not yet address the research question (“factors influencing return to work”). Because no analytical results are presented, the answer must be No. A short clarification that analytical findings will be reported in future papers would help.

11. Are the discussion and conclusions justified by the results? no

The discussion is minimal and cannot interpret findings in relation to the research question, because no analytical results are presented. The conclusions focus on future plans rather than findings. This is typical for a cohort profile, the discussion is not justified by results, because no results are provided.

12. Are the study limitations discussed adequately? no

The manuscript mentions some limitations (e.g., lack of comorbidity and lifestyle data), but the limitations section is brief and does not address: potential misclassification in register data, missing data handling, limitations of using SweJEM for exposure estimation, potential bias introduce by including individuals in both cohorts.

A more comprehensive limitations section would strengthen the manuscript.

Reviewer 2

Name **Stevens, Martin**

Affiliation **University of Groningen, Groningen, The Netherlands, Orthopedics**

Date **01-Jun-2026**

COI **None**

In my opinion your paper is a well written cohort profile. In fact I only have one minor suggestion.

- Although you mention the ICD-10-codes of the patients you included. You could consider to shortly describe more explicitly that only patients suffering from primary OA were included.

Reviewer **3**

Name **Ojeda-Thies, Cristina**

Affiliation **Hospital Universitario 12 de Octubre, Traumatology and Orthopaedic Surgery**

Date **15-Jun-2026**

COI **None**

This paper is a COHORT PROFILE. The manuscript is well presented and easily understandable. The region this study will be performed has unique characteristics that make this type of study particularly feasible, and the questions are pertinent and interest not only for Sweden. I believe the protocol presented is achievable and look forward to reading the results.

Some limitations the authors have not considered are sick leave for causes different than hip & knee arthroplasty, sick leave for periods shorter than 14 days, changes of work (type or demand).

Perhaps they could consider linkage with a dataset that measures use of medication.

VERSION 1 - AUTHOR RESPONSE

Reviewer: 1 – comments:
Dr. Bert Boonen, Zuyderland Medical Center
Comments to the Author:
Thank you for the opportunity to review this well constructed cohort profile. The manuscript presents a valuable and ambitious nationwide resource for studying sick leave and work disability following hip and knee arthroplasty in Sweden. The rationale is clear, the cohort is comprehensively described, and the linkage of multiple high quality national registers is a major strength. The paper is generally well written and easy to follow.

In addition to several minor suggestions for clarity, there are a few points where the reviewer form required a no response. These do not reflect major flaws but indicate areas where the manuscript could strengthened.

General comments
The manuscript is clear, well structured and presents a valuable nationwide cohort. A few areas could be refined for clarity and conciseness:

Authors' response
Thank you for your positive feedback and for your insightful suggestions, which have helped improve the clarity of our manuscript. We respond to each section below.

Reviewer: 1 – comments:
Abstract Well written; consider slightly shortening the Findings to date section to highlight only the key cohort characteristics.

Introduction Strong and well motivated; you may further emphasise the specific gaps in the existing literature on return to work.

Cohort description Comprehensive and transparent; a brief orienting summary at the start could improve readability.

Finding Informative but could be more concise to better match the purpose of a cohort profile.

Collaboration section Helpful, though it could be more specific regarding types of collaborations and include a clearer data availability statement.

Authors' response
Abstract: The Findings to date section has been shortened (page 2) *"The dataset includes patient and surgical characteristics, one-year patient-reported outcomes, first-line treatment participation, socioeconomic data, occupational exposures, and sick leave and disability pension data (five years before and after surgery). Data collection is complete."*

Introduction: Thank you for this helpful comment. We have now added the following sentences – marked in red (page 3-4): *"However, there is limited knowledge about the factors influencing RTW and sustained work participation following surgery [12]. For example, previous studies have demonstrated prolonged sickness absence and sex differences in RTW but have lacked detailed data on occupational exposures and disability pension [13]. Moreover, no nationwide study has examined longitudinal sick-leave trajectories and transitions to disability pension following arthroplasty. This knowledge gap may contribute to regional and provider-level variations in sick leave patterns."*

Cohort description: We have now added the following short summary in the beginning of the Cohort description section (page 4): *"The REWARD study comprises nationwide cohorts of individuals receiving hip and knee arthroplasty for osteoarthritis, along with matched reference populations."*

Findings: Thank you for your comment. We agree that the Findings section could be presented more concisely to align with the purpose of a cohort profile. However, we have chosen to retain the current level of detail, as we believe it provides important context for understanding the structure and characteristics of the cohort, including aspects such as re-surgeries and bilateral procedures, which have been highlighted as relevant for interpreting the dataset. We hope this rationale is acceptable.

Collaboration and data availability statement: These sections has now been rewritten for clarity as follows (page 16): "*We welcome collaboration on projects that align with the aims of the REWARD study, particularly those exploring cross-national comparisons of social insurance systems and return to work policies following joint arthroplasty, as well as methodological or outcome-focused analyses based on the cohort.*

Researchers interested in collaboration are invited to contact the principal investigator for the REWARD study (JW) to discuss potential projects or procedures for collaboration opportunities."

And:

"Data may be available upon reasonable request, subject to approval and appropriate legal agreements in accordance with Swedish and EU data protection legislation (e.g., GDPR). The dataset is governed by Umeå University, and cannot be made publicly available due to legal restrictions. Access may be granted for collaborative research projects following institutional and ethical approval. For further information regarding collaboration or data access, please contact the principal investigator (JW)."

Reviewer: 1 – comments:
With minor revisions to clarity and completeness, the manuscript will be well suited for publication.

Points marked no in the reviewer form
7. Statistics appropriate and fully described? no
The manuscript states that several analyses are planned but does not describe any statistical methods. For a cohort profile this is acceptable, but since the reviewer form asks specifically whether statistical methods are appropriate and fully described answer is No. The paper outlines what will be analysed but not how.
No statistical methods are described anywhere in the manuscript. Specifically, the manuscript does not indicate: how sick leave trajectories will be modelled, how determinants of return to work will be analysed, how confounding will be handled, how missing data will be addressed, whether time to event, mixed models, or trajectory modelling will be used, how comparisons with the reference cohort will be performed.

There is no Statistical analysis section at all. A brief outline of planned analytical approaches would address this.
9. Do the results address the research question? no
The manuscript presents only descriptive cohort characteristics. This is appropriate for a cohort profile, but it does not yet address the research question ("factors influencing return to work"). Because no analytical results are presented, the answer must be No. A short clarification that analytical findings will be reported in future papers would help.
11. Are the discussion and conclusions justified by the results? no
The discussion is minimal and cannot interpret findings in relation to the research question, because no analytical results are presented. The conclusions focus on future plans rather than findings. This is typical for a cohort profile, the discussion is not justified by results, because no results are provided.
12. Are the study limitations discussed adequately? no
The manuscript mentions some limitations (e.g., lack of comorbidity and lifestyle data), but the limitations section is brief and does not address: potential misclassification in register data, missing data handling, limitations of using SweJEM for exposure estimation, potential bias introduce by including individuals in both cohorts.
A more comprehensive limitations section would strengthen the manuscript.

Authors' response
Thank you for these comments. The manuscript is presented as a Cohort Profile and has been prepared in accordance with the journal´s Instruction for Authors, where detailed statistical methods and analytical results are not required. Since we currently are developing our initial analytical manuscript and continuously refining our approach as we become more familiar with the data, we consider it premature to provide detailed specifications at this stage, which also applies to the results and discussion sections.

We acknowledge that this limits the extent to which some reviewer form criteria can be fully addressed within this manuscript. Analytical findings will be reported in future study-specific publications, where we will ensure transparency and clarity in the reporting of methods and results.

We have, however, expanded the limitations section in response to your comments (page 15):
"Furthermore, limitations related to register-based data should be considered. Misclassification of exposures and outcomes may occur, as register data are not primarily collected for research purposes. For example, ICD-10 coding in the SSIA register is incomplete, limiting the ability to distinguish sick leave specifically related to OA or surgery. While subgroup analyses may be possible for individuals with recorded OA diagnoses, most analyses will focus on all-cause sick leave. In addition, sick leave for periods shorter than 14 days is not registered and will therefore not be possible to study. Missing data may also affect the analyses and should be addressed in the analyses.

In addition, the use of job-exposure matrices (JEMs), such as SweJEM, introduces exposure misclassification, as estimates are based on occupational groups rather than individual-level

measurements. JEMs are subject to Berkson-type measurement errors, which do typically not introduce systematic attenuation of estimated associations in linear analyses, unlike classical measurement error, but increased variance and wider confidence interval estimates. However, in non-linear models, such as Cox regression, some attenuation of effect estimates (bias toward the null) remains possible [22]. There is also a potential risk of bias related to cohort structure, as some individuals may contribute to both the hip and knee arthroplasty cohorts (e.g., if receiving both procedures), which will need to be carefully accounted for in future analyses."

Reviewer: 2
Dr. Martin Stevens, University of Groningen, Groningen, The Netherlands
Comments to the Author:
In my opinion your paper is a well written cohort profile. In fact I only have one minor suggestion.
- Although you mention the ICD-10-codes of the patients you included. You could consider to shortly describe more explicitly that only patients suffering from primary OA were included.

Authors' response and action
Thank you for your positive feedback on our manuscript. This is a relevant remark. We focused on primary OA of the hip and knee to maintain a more uniform study population, as other forms of OA (due to e.g. posttraumatic OA, dysplasia etc.) involve different underlying mechanisms, clinical trajectories and patient characteristics, which could introduce additional heterogeneity and complicate the interpretation of the results.

The following sentence has now been added under the heading Strengths and limitations (page 14): "*Only those with primary hip and knee OA were included to ensure a more homogeneous and interpretable study population."*

Reviewer: 3
Dr. Cristina Ojeda-Thies, Hospital Universitario 12 de Octubre
Comments to the Author:
This paper is a COHORT PROFILE. The manuscript is well presented and easily understandable. The region this study will be performed has unique characteristics that make this type of study particularly feasible, and the questions are pertinent and interest not only for Sweden. I believe the protocol presented is achievable and look forward to reading the results.

Some limitations the authors have not considered are sick leave for causes different than hip & knee arthroplasty, sick leave for periods shorter than 14 days, changes of work (type or demand).
Perhaps they could consider linkage with a dataset that measures use of medication.

Authors' response
Thank you for taking the time to review our Cohort profile and for your positive and constructive feedback.

Regarding linkage with dataset on the use of medication, since this manuscript was submitted, we have discussed this further within the research group and are now in the process of linkage data on comorbidity by ICD-10 codes as well as prescribed medications, to the REWARD research database (information added at page 12 and 14).

Regarding sick leave for other causes than hip and knee OA, we consider that this has already been highlighted as a limitation, and that our analyses most likely need to focus on all-cause sick leave: *"the inability to distinguish sick leave specifically related to OA or surgery, as ICD-10 coding in the SSIA register is incomplete. While subgroup analyses may be possible for individuals with recorded OA diagnoses, most analyses will focus on all-cause sick leave."*. However, we have added that we will not be able to study sick leave periods shorter than 14 days since those are not registered in the SSIA, as a limitation (added at page 14).

Regarding changes of work (type or demand), we are still exploring whether it is possible to capture and analyse these aspects. We believe the available data may allow this and therefore do not exclude it, but further consideration is needed to determine how and whether such analyses are feasible.
